# Frostbite treatment: a systematic review with meta-analyses

**DOI:** 10.1186/s13049-023-01160-3

**Published:** 2023-12-11

**Authors:** Ivo B. Regli, Rosmarie Oberhammer, Ken Zafren, Hermann Brugger, Giacomo Strapazzon

**Affiliations:** 1 Institute of Mountain Emergency Medicine, EURAC Research, Viale Druso 1, 39100 Bolzano, BZ Italy; 2 Dr. Regli’s Alpine Medical Services and Research, Unterägeri, ZG Switzerland; 3 Department of Internal and Emergency Medicine, Bürgerspital, Solothurn, SO Switzerland; 4 Department of Anesthesia and Intensive Care, Hospital of Brunico, Brunico, BZ Italy; 5 Department of Emergency Medicine, Alaska Native Medical Center, Anchorage, AK USA; 6 Department of Emergency Medicine, Stanford University Medical Center, Palo Alto, CA USA; 7 Department of Anesthesia and Intensive Care, Medical University Innsbruck, Innsbruck, Tyrol Austria

**Keywords:** Frostbite, Cold injury, Cold exposure systematic review, Meta-analysis, Thrombolysis, Iloprost, Mountain medicine, Wilderness medicine, Prostacyclin

## Abstract

**Introduction:**

Our objective was to perform a systematic review of the outcomes of various frostbite treatments to determine which treatments are effective. We also planned to perform meta-analyses of the outcomes of individual treatments for which suitable data were available.

**Main Body:**

We performed a systematic review and meta-analyses in accordance with the Preferred Reporting Items for Systematic Reviews and Meta-Analyses. We searched PubMed, Cochrane Trials, and EMBase to identify primary references from January 1, 1900, to June 18, 2022. After eliminating duplicates, we screened abstracts to identify eligible studies containing information on treatment and outcomes of Grade 2 to 4 frostbite. We performed meta-analyses of groups of articles that provided sufficient data. We registered our review in the prospective registry of systematic reviews PROSPERO (Nr. 293,693).

We identified 4,835 potentially relevant studies. We excluded 4,610 studies after abstract screening. We evaluated the full text of the remaining 225 studies, excluding 154. Ultimately, we included 71 articles with 978 cases of frostbite originating from 1 randomized controlled trial, 20 cohort studies and 51 case reports. We found wide variations in classifications of treatments and outcomes. The two meta-analyses we performed both found that patients treated with thrombolytics within 24 h had better outcomes than patients treated with other modalities. The one randomized controlled trial found that the prostacyclin analog iloprost was beneficial in severe frostbite if administered within 48 h.

**Conclusions:**

Iloprost and thrombolysis may be beneficial for treating frostbite. The effectiveness of other commonly used treatments has not been validated. More prospective data from clinical trials or an international registry may help to inform optimal treatment.

**Supplementary Information:**

The online version contains supplementary material available at 10.1186/s13049-023-01160-3.

## Background

Frostbite is a local tissue injury caused by cold exposure with freezing [1, 2]. After tissue reaches subfreezing temperatures, intra- and extracellular ice formation cause electrolyte and pH shifts as well as cell membrane disruption, resulting in cell death and tissue destruction [3]. When tissue rewarms, reperfusion injury can cause inflammation, vasoconstriction, thrombus formation, endothelial damage, edema, and ischemia with further tissue damage [4]. Clinically, frostbite presents with a wide spectrum of injury ranging from no loss of tissue to extensive necrosis requiring amputations [1, 2].

Regardless of treatment, meticulous wound care with delayed debridement is critical [5]. Acute treatment often aims to reverse vasoconstriction and thrombosis to limit progression of injury. Although there is only one published randomized controlled trial studying the relative effectiveness of various frostbite treatments, there are many retrospective studies, case reports, and case series, reporting the results of various treatments with varying rates of tissue salvage [6, 7]. Pharmacologic treatment can use thrombolytics, such as recombinant tissue plasminogen activator (tPA), vasodilators, such as iloprost, a systemic prostacyclin analog, [8–10] and nifedipine, as well as phosphodiesterase inhibitors, such as sildenafil [7]. Regional anesthesia with peripheral nerve blocks [11], surgical sympathectomies [12] and colloid infusions [13] have also been used for their vasodilatory effects. Adjunctive pharmacologic treatments can include cyclooxygenase inhibitors, such as acetylsalicylic acid (ASA), and ibuprofen [5, 14], platelet inhibitors such as clopidogrel [15], anticoagulation with heparin or low-molecular weight heparin (LMWH), [16, 17] and hyperbaric oxygen therapy (HBOT) [18]. Medications for pain relief include opiates and other drugs without significant anti-inflammatory effect [5, 7].

Thus far, no study has systematically evaluated the outcome of frostbite treatment based on different treatment strategies. Here, we aimed to systematically evaluate the accessible literature regarding the effectiveness of various approaches to treating frostbite. Where sufficient data existed, we compiled meta-analyses for individual treatment methods.

## Methods

**Fig. 1:**
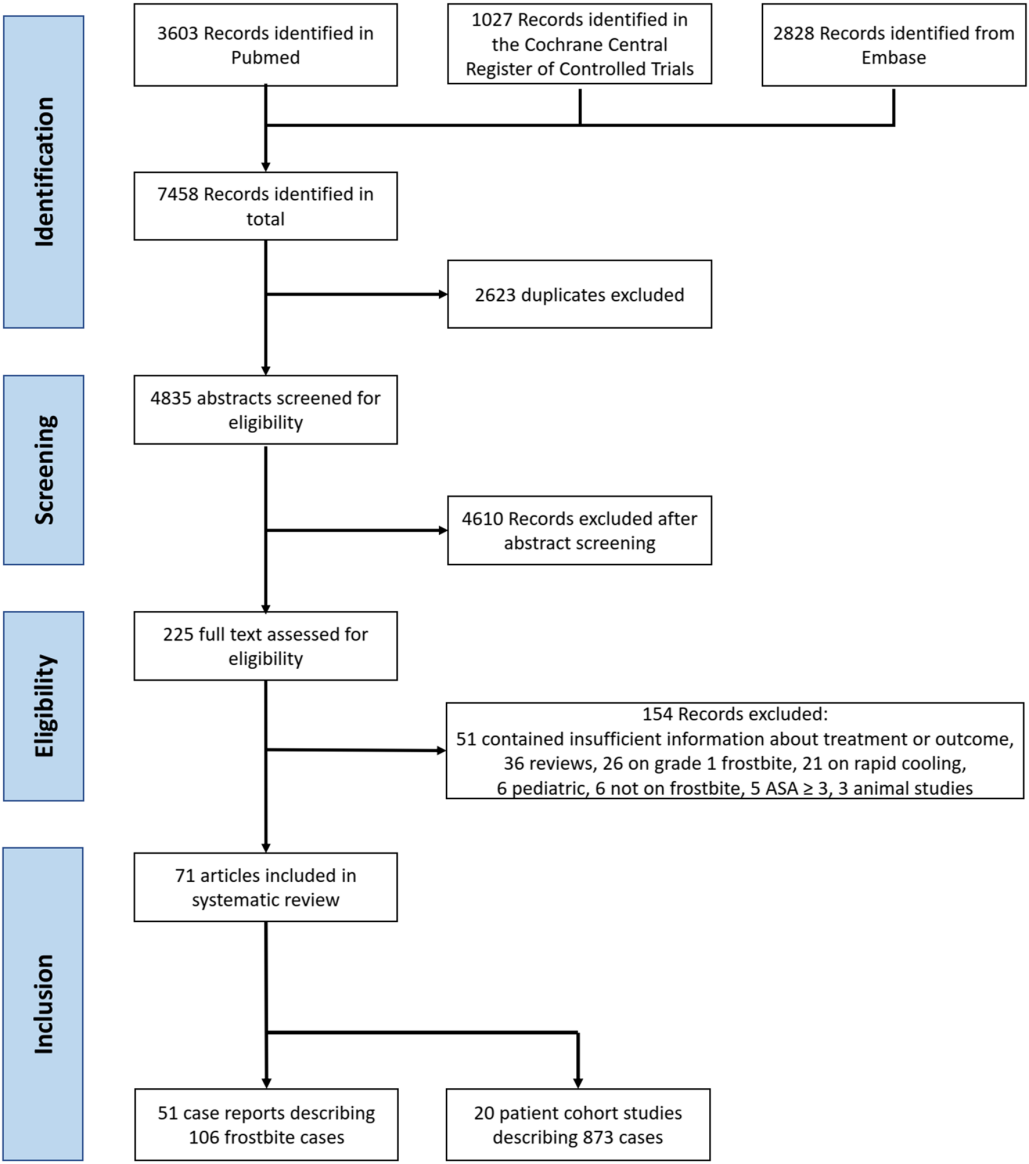
Article selection process flow chart. ASA: American Society of Anesthesiologists Physical Status Classification System

We performed a systematic review and meta-analyses in accordance with the Preferred Reporting Items for Systematic Reviews and Meta-Analysis (PRISMA—www.prisma-statement.org). We registered our review in the prospective registry of systematic reviews, PROSPERO (293,693) [19]. Figure 1 shows the article selection process.

### Search strategy, and study selection

We used PubMed (search terms "Cold Injury"[Mesh] OR frostbit*[tiab] OR cold injur*[tiab]), Cochrane Trials (frostbit*:ab,ti,kw OR cold injur*:ab,ti,kw OR MeSH descriptor: [Cold Injury] explode all trees), and EMBase (frostbit*:ab,ti OR 'cold injur*':ab,ti) to identify primary references from January 1, 1900 to June 18, 2022. After elimination of duplicates, we reviewed the citations for eligible studies selecting studies based on the abstracts. We then read the full texts of the selected studies to identify those suitable for inclusion in the review. We assessed methodologic quality using the ROBIS tool [20] to minimize risk of bias.

### Eligibility criteria

We identified studies that contained information about treatments and outcomes of Grade 2 to 4 frostbite injuries [21]. We included only articles in which specific outcomes for specific treatments could be determined. We screened articles in English, French, German, Italian, and Spanish. We excluded data from patients with severe comorbidities (American Society of Anesthesiologists Physical Status Class ≥ 3) [22]. Although the ASA classification is typically used for preoperative evaluation, we used the system to exclude patients with comorbidities that could affect frostbite treatment outcomes. We also excluded reports of patients with flash freezing injuries caused by accidental skin exposure to cryogenic materials, such as dry ice and liquid nitrogen. We excluded data from pediatric cases (< 18 years of age) if age was reported. In two studies, it was not possible to exclude pediatric data [23, 24]. We excluded abstracts, presentations, conference proceedings, and reviews.

### Data extraction and classification

One reviewer (IR) extracted the data from the selected studies. For each study the following information was extracted and summarized: sex, age, body part(s) involved, frostbite grade [21], prehospital treatment, in-hospital treatment, and outcome.

We classified frostbite using the system described by Cauchy et al. in 2001 [21]. It was a challenge to synthesize data from a century of literature with a diverse array of frostbite classifications. We attempted to ensure consistency by applying this uniform classification to earlier studies and to studies that used alternative classifications, classifying frostbite cases based on the published images and descriptions.

We focused on treatments that might influence the pathogenesis of frostbite: vasodilation (iloprost, sympathectomy, and colloid solution infusion), inhibition of platelet aggregation (ASA, ibuprofen, and clopidogrel), anticoagulation (heparin and LMWH), thrombolysis (tPA), and optimization of tissue oxygenation (HBOT). We classified interventions such as wound care and analgesic treatment with opiates and other drugs without significant anti-inflammatory effects as conservative treatment.

### Data analysis

We used multiple methods to quantify the outcomes of frostbite treatments, because there was significant heterogeneity of data reporting among the studies.

#### (Modified) Hennepin Score

The Hennepin Score is primarily used for research purposes, offering an objective means of evaluating frostbite severity and facilitating outcome comparisons across various studies [25]. A numeric value is assigned to each frostbitten phalanx, digit, toe, and limb at risk, as indicated by low or no perfusion on a triple-phase bone scan (99mTc scintigraphy). This defines the tissue-at-risk score (R). If an article did not report this method, we based R on a modified Hennepin Score by assessing the clinical appearance of the extremities. We then estimated the amputation score (A), using the same method. The difference between R and A is the tissue salvage score: (S): S = R – A. The ratio between S and R gives the tissue salvage rate (TSR), expressed as a percentage (S / R × 100).

#### Digit salvage rate

The digit salvage rate uses the ratio of the digits at risk (DR] and digits amputated (DA] expressed as a percentage: (1-(DA/DR) × 100) [8].

#### Phalanx salvage rate

The phalanx salvage rate uses the ratio of the phalanges at risk (PR) and the phalanges amputated (PA) expressed as percentage: (1-PA/PR) × 100 [26].

#### Amputation score

Whenever other metrics were not applicable or not available to quantify frostbite treatment outcomes, we analyzed the frequency of cases resulting in amputation. In this approach, treatment success is evaluated by monitoring the number of patients who ultimately undergo amputation as a result of frostbite compared to the total number of patients with frostbite in a given study. Although it does not provide as much detail as other metrics, it still provides insight into the effectiveness of various treatments.

### Statistical analysis

When feasible, we conducted meta-analyses to evaluate various treatments using both fixed effects and random effects models. This was achievable when at least two studies examined similar treatments (for instance, administering the same substance in the intervention group) in patient populations with comparable characteristics. In the first meta-analysis, we analyzed the salvage rate using the Hennepin Score. We estimated the between-study variance (τ^2^) using the DerSimonian-Laird method. In the second meta-analysis, we analyzed the risk ratio of amputation probability of a digit at risk, estimating τ^2^ using the Paule-Mandel method. We estimated heterogeneity using the statistic *I*^*2*^. We used R version 4.0.4 statistical software with meta and metasens libraries for these analyses [27, 28].

## Results

After eliminating duplicate entries, we found 4,835 studies that were potentially relevant (Fig. 1). We then assessed the abstracts excluding 4,610 articles, primarily for lack of relevance or insufficient data. We obtained the full texts of the remaining 225 studies for further evaluation. We rejected 154 studies that failed to meet our eligibility criteria, primarily because they lacked adequate information about treatments or outcomes. Our selection procedure led to the inclusion of 978 frostbite cases from the remaining 71 articles.

Table 1 displays the baseline demographics, with men representing 78% of the cases, women 17%, and 5% not specifying sex. Upper and lower extremities were affected equally. A total of 873 cases were from studies that reported treatments across different patient cohorts (Additional file 1: eTables 2–5), while the remaining 105 cases were reported in 51 case reports and case series (Additional file 1: eTable 1).

**Table 1:** Baseline patient characteristics. The number of cases, age, sex and affected body part of the patients in the articles included are shown. The cases are arranged into the groups all cases, individual cases from case reports and series, and patient cohort cases

	All cases	Individual cases	Patient cohort cases
Cases	978	105	873
Age	Mean: 41 years^a^	Mean: 42 years	Mean: 41 years^a^
		Median: 42 years	
Sex	Female: 169 (17%)	Female: 11 (10%)	Female: 158 (18%)
	Male: 760 (78%)	Male: 72 (69%)	Male: 688 (79%)
	Not specified: 49 (5%)	Not specified: 22 (21%)	Not specified: 27 (3%)
Affected body part(s)	Upper extremities: 209 (21%)	Upper extremities: 42 (40%)	Upper extremities: 167 (19%)
	Lower extremities: 204 (21%)	Lower extremities: 53 (50%)	Lower extremities: 151 (17%)
	Upper and lower extremities: 193 (20%)	Upper and lower extremities: 8 (8%)	Upper and lower extremities: 185 (21%)
	Not specified: 372 (38%)	Not specified: 2 (2%)	Not specified: 370 (43%)

^a^4 articles, with 226 patients, were excluded because the mean age could not be calculated

Table 2 shows the treatment outcomes of cohort studies, grouped according to the methods used to quantify the outcomes of different frostbite treatments. Table 2 presents the treatment outcomes from cohort studies, organized according to the methods employed to measure the outcomes of various frostbite treatments. Patients receiving thrombolysis, iloprost, or a combination experienced higher tissue and digit salvage rates than other patients.. The Yukon frostbite protocol led to an increased digit salvage rate (overall 80%, with 100% for grade 2 -3 and 50% for grade 4 frostbite) compared to historical controls (31% for grade 2, 67% for grade 3 and 98%–100% for grade 4 frostbite) in patients with grade 2–4 frostbite treated with iloprost and patients with grade 4 frostbite who also received tPA and heparin [10, 21].

**Table 2:** Outcomes of frostbite treated with different modalities in patient cohorts. Treatments are grouped according to the quantification methods. HBOT = Hyperbaric oxygen therapy

Outcome quantified by the Hennepin Score (3 studies) ^a^[9, 24, 31]		
Treatment	Cases	Tissue salvage rate
Treatment including thrombolysis	78	76%
Treatment without thrombolysis	45	49%

Outcome quantified by the digit salvage rate (10 studies)^a^[8–10, 18, 23, 30, 32, 48–50]		
Treatment	Cases	Digit salvage rate
Treatment including thrombolysis	115	69%
Treatment including iloprost + HBOT	28	98%
Treatment including iloprost	30	85%
Yukon frostbite protocol ^b^	22	80%
Treatment without thrombolysis, iloprost or HBOT	35	48%

Outcome quantified by the phalanges salvage rate (1 study) [26]		
Treatment	Cases	Phalanges salvage rate
Treatment including thrombolysis	5	87%

Outcome quantified by the amputation rate (7 studies) [29, 51–56]			
Treatment	Cases	No amputation (%)	Amputation (%)
Treatment including thrombolysis	91	64 (70%)	27 (30%)
Treatment including HBOT	22	14 (64%)	8 (36%)
Treatment without thrombolysis and HBOT	364	188 (52%)	176 (48%)

^a^One article was **included** in two tables [9]

^b^Data is **represented** as outcome of the protocol. [10]

Among the 20 studies in our review, only seven included more than one treatment modality [8, 24, 26, 29–32]. Six studies compared patients treated with and without thrombolysis.

**Fig. 2:**
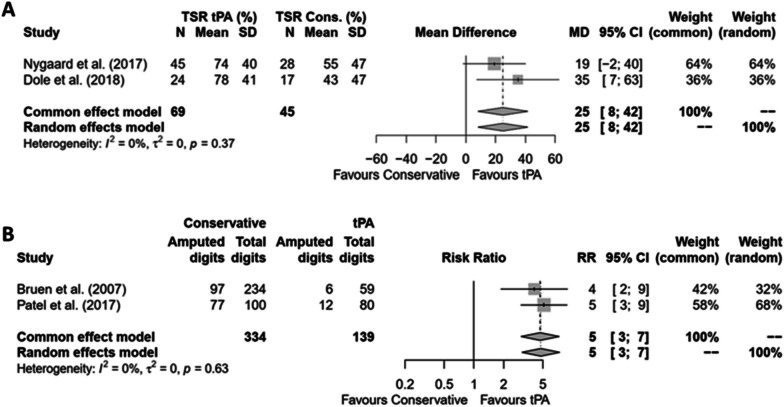
Meta-analyses of studies comparing thrombolysis and conservative treatment. Mean difference of the salvage rate of the Hennepin Frostbite Score (**A**) and risk ratio of the amputation probability of a digit at risk (**B**). The between-study variance τ^2^ was estimated by means of DerSimonian-Laird (A) and Paule-Mandel (**B**) methods. P-value refers to the heterogeneity test. I^2^, heterogeneity statistic; τ^2^, between-study variance; CI, confidence interval; MD, mean difference; N, numerosity; RR, risk ratio; SD, standard deviation; SR, salvage rate; tPA, tissue plasminogen activator

We conducted two meta-analyses. The first used the two studies that measured treatment outcomes with the Hennepin Score (Tissue Salvage Rate (TSR)) (Fig. 2), [24, 31]. The second used two studies that calculated digit salvage rates (DSR) as outcome measures [30, 32]. In both cases, thrombolytics significantly improved tissue (Fig. 2A) or digit (Fig. 2B) salvage rates, regardless of whether a common effects model or a random effects model was applied. The first meta-analysis reported a mean TSR difference of 25% (95% CI 8% – 42%) for both models, while the second showed a risk ratio of 5 (95% CI 3 – 7) for both models. Two studies allowed only binary distinctions of treatment success (amputated or not amputated) [26, 29] A combined meta-analysis was not feasible because of differences in patient populations (grade 2 vs. grade 3–4 frostbite).

Table 3 shows the number of individual cases for each treatment. Because of variations in treatments among articles, there were generally few articles for each treatment. The only published prospective, controlled, randomized study [8] reported significantly better outcomes for patients treated with iloprost. This was a small study (*n* = 47) (Table 4).

**Table 3:** Frostbite case reports with treatments

Treatment	Cases	References
Thrombolysis + heparin	4	[57, 58]
Thrombolysis + heparin + ASA	1	[59]
Thrombolysis + heparin + papaverin	1	[60]
Thrombolysis + HBOT	1	[61]
Thrombolysis + HBOT + abciximab	1	[62]
Iloprost	1	[63]
Iloprost + ASA + LMWH	1	[64]
Iloprost + ASA	7	[64–66]
Iloprost + ASA + LMWD	1	[17]
Iloprost + LMWH	1	[17]
Iloprost + Ibuprofen	2	[67, 68]
HBO	4	[69–71]
HBOT + Iloprost	1	[72]
HBOT + pentoxifylline + Ibuprofen + aloe vera	1	[73]
HBOT + pentoxifylline + LMWH	2	[74]
Regional anesthesia	2	[75, 76]
Regional anesthesia + heparin + Phenindione	3	[77]
Regional anesthesia + dexketoprofene	1	[11]
Regional pharmacological sympathectomy + LMWD	1	[78]
Stellate ganglion blocks	1	[50]
Surgical sympathectomy	21	[12]
Ibuprofen	3	[79–81]
Ibuprofen + heparin	1	[82]
Ibuprofen + aloe vera	1	[68]
Ibuprofen + nifedipine + alprostadil	1	[83]
ASA	1	[84]
ASA + LMWD	1	[85]
ASA + LMWH	1	[86]
ASA + LMWH + pentoxifylline	1	[87]
ASA + heparin	1	[88]
LMWD	2	[87, 90]
Bradykinin	2	[91]
Reserpine	4	[92, 93]
ACTH	2	[94]
2.45 GHz microwaves + heparin	1	[95]
Conservative treatment	27	[96–104]

ACTH = Adrenocorticotropic hormone, ASA = Acetylsalicylic acid, HBOT = Hyperbaric oxygen therapy, LMWD = Low molecular weight dextran, LMWH = Low molecular weight heparin

**Table 4:** Results of the prospective randomized controlled study 8

The prospective randomized controlled study by Cauchy et al. 8			
Treatment	Cases	Digit salvage rate	p-value
Buflomedil + ASA	15	60%	
Buflomedil + Iloprost + ASA	16	100%	< 0.001
Buflomedil + tPA + Iloprost + ASA	16	97%	< 0.03

The p-value refers to the comparison with the Buflomedil alone group by Fisher’s exact test. tPA = Tissue plasminogen activator, ASA = Acetylsalicylic acid

## Discussion

We found considerable variability in treatments and outcome classifications. We were often only able to describe outcome data instead of reporting quantitative results. Also, older treatments such as surgical sympathectomy have become obsolete with the advent of newer regional anesthesia techniques such as peripheral nerve blocks for temporary pharmacologic sympatholysis.

Our results suggest that thrombolysis or intravenous iloprost is effective when administered promptly to treat severe frostbite. For grade 3–4 frostbite the Wilderness Medical Society frostbite guidelines recommend the use of intravenous iloprost within 48 h of injury, and thrombolysis within 24 h of injury [5]. The Helsinki protocol recommends the use of tPA for patients with grade 3–4 frostbite presenting within 48 h of injury with angiographic evidence of thrombosis [9]. Patients with contraindications to thrombolysis (platelet count < 100 × 10^9^/L, hematocrit < 30%), signs of vasospasm on angiography, or poor response to thrombolysis should be treated with iloprost. A retrospective analysis found an 81% tissue salvage rate using the Hennepin score for 20 patients with grade 3–4 frostbite treated using the Helsinki protocol [9]. The Yukon protocol recommends treatment of patients with grade 3–4 frostbite presenting within 72 h of injury with iloprost [10]. Patients with grade 4 frostbite presenting within 24 h of injury, should also receive tPA.

Iloprost is a synthetic prostaglandin I2 that has been used to treat frostbite [33]. Like other prostacyclins, it inhibits platelet aggregation and promotes vasodilation [34]. Iloprost may stimulate the release of endogenous tissue plasminogen activator or counteract its inhibitory effects [35]. Iloprost reduces vasoconstriction induced by thromboxane A2 [36], and may reduce oxidative stress from free radicals, moderating reperfusion injury [37, 38]. The effect on platelet aggregation may be reversed within two hours), but prostacyclin effects may disrupt the vicious cycle of activated platelets and leukocytes that damages endothelium [35, 39].

Thrombolytics work by binding to fibrin within a thrombus and activating plasminogen, causing local fibrinolysis and inhibiting blood clot formation. [40] A systematic review found comparable limb salvage rates for frostbite patients treated with intra-arterial and intravenous thrombolysis, (76% vs. 77%) [41]. Use of thrombolytics is associated with a risk of hemorrhage. A study of bleeding complications in patients with severe frostbite treated using intravenous tPA found that 8% of patients developed bleeding that necessitated changes in management [42].

Recommendations to treat of frostbite with low molecular weight dextran, ibuprofen, or topical aloe vera are based on mechanistic reasoning or animal studies, rather than clinical data. The limited number of reports on these treatments precluded us from drawing conclusions about their clinical effectiveness. Low molecular weight dextran, thought to reduce blood viscosity and inhibit thrombus formation, has demonstrated decreased necrosis in animal models of frostbite [13, 43, 44], but is not available in many countries. Nonsteroidal anti-inflammatory drugs (NSAIDs) block the effects of cyclo-oxygenases, decreasing the production of prostaglandins and thromboxane, mediators that can cause increased vasoconstriction, ischemia, and inflammatory tissue damage [3, 45]. An experimental rabbit frostbite model showed improved tissue survival in animals treated with ASA. [14] Because aspirin causes irreversible inhibition of cyclooxygenase that could disrupt wound healing, some authors have suggested that ibuprofen might be preferred as a treatment for frostbite [5, 46]. There are no head-to-head comparison studies. Topical aloe vera reduces formation of prostaglandins and thromboxane and has been shown to increase tissue survival in an experimental frostbite rabbit model [14], but has not been studied in humans.

A study of patients with grade 3–4 frostbite treated with HBOT, aspirin, and iloprost suggested a possible benefit of HBOT [18]. This multicenter prospective single-arm study compared outcomes with those of a historical cohort treated with aspirin and iloprost, but not HBOT and found that the group receiving HBOT had a significantly higher number of preserved tissue segments per patient. The reduction in amputation rates was also more pronounced in patients treated with HBOT.

Theoretically, hypobaric hypoxia at high altitudes might exacerbate the risk and severity of frostbite. A retrospective study reported that frostbite severity increased disproportionately at altitudes above 5,200 m [47]. These data are confounded by colder temperatures and stronger winds at higher elevations.

Among the various methods to measure frostbite injuries, we regard the Hennepin score as the most precise. The original report of the Hennepin score found a negative correlation of S and R (correlation coefficient, − 0.14, *p* = 0.001) in frostbite patients who underwent Tc-99 m three-phase bone scans of the affected extremities, with high consistency between evaluators (correlation coefficient: 0.93) [25].

## Limitations

The reported treatments exhibited considerable variations with a wide array of medications administered at different doses and frequencies. The studies were heterogeneous, with various frostbite classification systems. Our retrospective classification may have introduced errors.

Many studies were low quality. There was only one randomized controlled trial. Direct medication comparisons were scarce. There was only one multicenter study. Our analysis could not account for coexisting injuries or conditions such as trauma, volume depletion, or hypothermia. We were also unable to control for other potential confounders, such as mechanical damage to the injury site, the method of rewarming and the quality of wound care.

## Conclusions

Iloprost and thrombolysis may be beneficial for treating frostbite. The effectiveness of other commonly used treatments has not been validated. More prospective data from clinical trials or international registry may help to inform optimal treatment. Because there is a low incidence of severe frostbite at any single institution, conducting multicenter trials, or establishing international registries could help reach higher levels of evidence.

### Supplementary Information

**Additional file 1. eTable 1: **List of case reports. **eTable 2:** Patient cohort studies with outcomes quantified by the Hennepin score. **eTable 3:** Patient cohort studies with outcomes quantified by digit salvage rate. **eTable 4:** Patient cohort studies with outcomes quantified by phalanx salvage rate. **eTable 5:** Patient cohort studies with outcomes quantified by amputations.

## Data Availability

Data derived from existing literature can be found within the main article as well as in the supplementary tables. The source data can be obtained from the respective publications cited in the reference section.

## Abbreviations

ASA: Acetylsalicylic acid
CI: Confidence interval
DA: Digits amputated
DR: Digits at risk
DSR: Digit salvage rate
HBOT: Hyperbaric oxygen therapy
LMWH: Low-molecular weight heparin
NSAIDs: Nonsteroidal anti-inflammatory drugs
PA: Phalanges amputated
PR: Phalanges at risk
PRISMA: Preferred reporting items for systematic reviews and meta-analysis
PROSPERO: Prospective registry of systematic reviews
RCT: Randomized controlled trial
ROBIS: Risk of bias in systematic reviews
tPA: Tissue plasminogen activator
TSR: Tissue salvage rate
