# Supplementary material for: Frostbite treatment: a systematic review with meta-analyses
Source: Scand J Trauma Resusc Emerg Med. 2023 Dec 11;31:96. doi: 10.1186/s13049-023-01160-3 (PMC10712146; doi:10.1186/s13049-023-01160-3)
Supplement: Supplementary file 1 — Additional file 1. eTable 1: List of case reports. eTable 2: Patient cohort studies with outcomes quantified by the Hennepin score. eTable 3: Patient cohort studies with outcomes quantified by digit salvage rate. eTable 4: Patient cohort studies with outcomes quantified by phalanx salvage rate. eTable 5: Patient cohort studies with outcomes quantified by amputations. [file 13049_2023_1160_MOESM1_ESM.docx]

**eTable 1: List of case reports**

[R] = tissue at risk score, [A] = tissue amputated score, [S] = [R]-[A] = Tissue Salvage Score, Tissue Salvage rate = ([S] / [R]*100), ACTH = Adrenocorticotropic hormone, ASA = Acetylsalicylic acid, LMWD = Low molecular weight dextran, LMWH = Low molecular weight heparin, tPA = Tissue plasminogen activator, HBOT= Hyperbaric oxygen therapy

| **Article** | **Age** | **Sex** | **Extremity involved** | **Treatment** | **[R]** | **[A]** | **[S]** | **Tissue Salvage Rate** | **Highest**  **Grade** |
| --- | --- | --- | --- | --- | --- | --- | --- | --- | --- |
| Ali et al. 1982 (77) | 20 | Male | Upper | Stellate ganglion block with bupivacaine | 4 | 0 | 4 | 100% | 3 |
| Aygit et al., 2002 (87) | 34 | Male | Lower | ASA + LMWD | 40 | 0 | 40 | 100% | 3 |
| Banzo et al., 2002 (101) | 38 | Male | Upper and lower | Conservative treatment | 2.25 | 2.25 | 0 | 0% | 2 |
| Barker et al., 1997 (71) | 55 | Male | Lower | HBOT | 40 | 40 | 0 | 0% | 4 |
| Barker et al., 1997 (71) | 28 | Male | Upper | HBOT | 20 | 20 | 0 | 0% | 3 |
| Brandão et al., 2018 (106) | 27 | Male | Lower | Conservative treatment | 1 | 0 | 1 | 100% | 3 |
| Brown et al., 1986 (100) | 20 | Male | Lower | Conservative treatment | 24 | 14 | 10 | 42% | 3 |
| Campbell et al., 1961 (79) | 47 | Male | Lower | Epidural mepivacaine + heparin | 2.75 | 1 | 1.8 | 64% | 3 |
| Campbell et al., 1961 (79) | 47 | Male | Lower | Epidural mepivacaine + heparin | 10 | 6 | 4 | 40% | 3 |
| Daniel et al., 2022 (99) | 34 | Male | Lower | Conservative treatment | 20 | 0 | 20 | 100% | 3 |
| Dwivedi et al., 2015 (75) | 22 | Male | Lower | HBOT+ aloe vera + pentoxifylline + ibuprofen | 0.75 | 0 | 0.8 | 100% | 2 |
| Ekdahl et al., 2017 (81) | 28 | Male | Upper | Ibuprofen | 4 | 0 | 4 | 100% | 3 |
| Engkvist, 1985 (80) | 22 | Male | Lower | LMWD, regional intravenous guanethidine block | 25 | 0 | 25 | 100% | 3 |
| Erba et al., 2011 (88) | 41 | Male | Lower | ASA, LMWH | 3 | 1 | 2 | 67% | 3 |
| Erikson et al., 1974 (93) | 46 | Male | Lower | Bradykinin | 3 | 0,5 | 2.5 | 83% | 2 |
| Erikson et al., 1974 (93) | 53 | Male | Upper | Bradykinin | 22.5 | 19 | 3.5 | 16% | 3 |
| Ezquerra-Herrardo et al., 2013 (98) | 42 | Male | Upper and lower | Conservative treatment | 35 | 33 | 2 | 6% | 4 |
| Folio et al., 2007 (72) | 28 | Female | Upper | HBOT | 20 | 0 | 20 | 100% | 3 |
| Gavrilin et al., 2021(97) | 27 | Male | Upper and lower | 2.45 GHz Microwaves + heparin | 20 | 0 | 20 | 100% | 4 |
| Glenn et al., 1952 (96) | 57 | Male | Lower | ACTH | 25 | 0 | 25 | 100% | 3 |
| Glenn et al., 1952 (96) | 40 | Male | Lower | ACTH | 25 | 0 | 25 | 100% | 3 |
| Goertz et al., 2011 (84) | 36 | Female | Upper | Ibuprofen, heparin | 2 | 0 | 2 | 100% | 2 |
| Gralino et al., 1976 (94) | 30 | Male | Lower | Reserpine | 36 | 0 | 36 | 100% | 4 |
| Gralino et al., 1976 (94) | 59 | Male | Lower | Reserpine | 36 | 0 | 36 | 100% | 4 |
| Gross et al., 2012 (61) | 59 | Female | Upper | tPA + heparin + ASA | 20 | 12 | 8 | 40% | 3 |
| Hidgon et al., 2015 (64) | 33 | Male | Upper | tPA + abciximab + HBOT | 14 | 0.5 | 14 | 96% | 3 |
| Hödl et al., 2005 (17) | 18 | Male | Upper | LMWD + ASA | 0.25 | 0 | 0.3 | 100% | 2 |
| Hödl et al., 2005 (17) | 19 | Male | Lower | Iloprost + LMWD+ ASA | 20 | 2 | 18 | 90% | 3 |
| Hödl et al., 2005 (17) | 55 | Male | Lower | Iloprost + LMWH | 6 | 6 | 0 | 0% | 3 |
| Ibrahim et al., 2015 (59) | 29 | Male | Lower | tPA + heparin | 10 | 0 | 10 | 100% | 3 |
| Ibrahim et al., 2015 (59) | 52 | Male | Lower | tPA + heparin | 10 | 0 | 10 | 100% | 3 |
| Ibrahim et al., 2015 (59) | 58 | Male | Lower | tPA + heparin | 6.5 | 0 | 6.5 | 100% | 3 |
| Irarrázaval et al., 2018 (69) | 60 | Male | Upper | Iloprost + ibuprofen | 7 | 3.5 | 3.5 | 50% | 3 |
| Jud et al., 2019 (66) | 45 | Male | Upper | Iloprost + ASA | 3 | 0 | 3 | 100% | 3 |
| Kayser et al., 1993 (91) | 19 | Male | Upper and lower | LMWD | 40 | 40 | 0 | 0% | 4 |
| Johnson-Arbor et al., 2014 (107) | 42 | Male | Upper | Conservative treatment | 18 | 2 | 16 | 89% | 3 |
| Joseph et al., 2020 (60) | 36 | Male | Upper | tPA + heparin | 11 | 0 | 11 | 100% | 3 |
| Kemper et al., 2014 (73) | 32 | Female | Lower | HBOT | 0.5 | 0.5 | 0 | 0% | 2 |
| Kroeger et al., 2004 (85) | 32 | Female | Lower | Ibuprofen, nifedipine, alprostadil | 10 | 6 | 4 | 40% | 3 |
| Lansdorp et al., 2017 (76) | 58 | Female | Lower | HBOT+ pentoxifylline + LMWH | 10 | 0,5 | 9.5 | 95% | 3 |
| Lansdorp et al., 2017 (76) | 62 | Male | Lower | HBOT+ pentoxifylline + LMWH | 25 | 20 | 5 | 20% | 3 |
| Lorentzen et al., 2018 (82) | 53 | Male | Upper | Ibuprofen | 20 | 0 | 20 | 100% | 4 |
| MacLennan et al., 2021 (65) | 48 | Male | Lower | Iloprost | 1 | 0 | 1 | 100% | 2 |
| Masters et al., 2018 (63) | 24 | Male | Upper and lower | tPA + HBOT | 25 | 2.5 | 23 | 90% | 3 |
| Magnan et al., 2022 (74) | 36 | Male | Upper | Iloprost + HBOT | 20 | 0 | 20 | 100% | 3 |
| Mulgrew et al., 2013 (102) | 24 | Male | Lower | Conservative treatment | 8 | 0 | 8 | 100% | 3 |
| Page et al.,  1983 (105) | 27 | Female | Upper | Conservative treatment | 8 | 0.5 | 7.5 | 94% | 3 |
| Page et al.,  1983 (105) | 49 | Male | Upper | Conservative treatment | 5 | 0,5 | 4.5 | 90% | 3 |
| Page et al.,  1983 (105) | 30 | Male | Upper | Conservative treatment | 4.5 | 1.5 | 3 | 67% | 2 |
| Page et al.,  1983 (105) | 54 | Not specified | Upper | Conservative treatment | 8 | 1.5 | 6.5 | 81.% | 3 |
| Page et al.,  1983 (105) | 20 | Male | Upper | Conservative treatment | 8 | 1 | 7 | 88% | 3 |
| Page et al.,  1983 (105) | 73 | Male | Upper | Conservative treatment | 5 | 2 | 3 | 60% | 3 |
| Page et al.,  1983 (105) | 31 | Male | Upper | Conservative treatment | 10 | 1 | 9 | 90% | 3 |
| Page et al.,  1983 (105) | 72 | Male | Upper | Conservative treatment | 8 | 2.5 | 5.5 | 69% | 3 |
| Page et al.,  1983 (105) | 63 | Female | Upper | Conservative treatment | 17 | 4.5 | 13 | 74% | 3 |
| Page et al.,  1983 (105) | 32 | Male | Upper | Conservative treatment | 20 | 1.5 | 19 | 93% | 3 |
| Page et al.,  1983 (105) | 77 | Male | Upper | Conservative treatment | 10 | 4 | 6 | 60% | 3 |
| Page et al.,  1983 (105) | 60 | Male | Upper | Conservative treatment | 20 | 4.5 | 16 | 78% | 3 |
| Page et al.,  1983 (105) | 60 | Male | Upper | Conservative treatment | 20 | 3.5 | 17 | 83% | 3 |
| Page et al.,  1983 (105) | 65 | Male | Upper | Conservative treatment | 20 | 3 | 17 | 85% | 3 |
| Page et al.,  1983 (105) | 46 | Female | Upper | Conservative treatment | 16 | 4 | 12 | 75% | 3 |
| Page et al.,  1983 (105) | 59 | Male | Upper | Conservative treatment | 25 | 19 | 6 | 24% | 4 |
| Page et al.,  1983 (105) | 27 | Male | Upper | Conservative treatment | 12.5 | 5.5 | 7 | 56% | 4 |
| Page et al.,  1983 (105) | 67 | Male | Upper | Conservative treatment | 25 | 20 | 5 | 20% | 4 |
| Page et al.,  1983 (105) | 20 | Male | Upper | Conservative treatment | 30 | 30 | 0 | 0% | 4 |
| Pandey et al., 2018 (68) | 49 | Male | Upper | Iloprost | 3.75 | 1.5 | 2.3 | 60% | 3 |
| Pandey et al., 2018 (68) | 38 | Male | Lower | Iloprost | 1.5 | 0 | 1.5 | 100% | 2 |
| Pandey et al., 2018 (68) | 21 | Male | Lower | Iloprost | 10 | 0 | 10 | 100% | 3 |
| Pandey et al., 2018 (68) | 54 | Male | Lower | Iloprost | 4 | 2 | 2 | 50% | 3 |
| Pandey et al., 2018 (68) | 33 | Male | Upper and lower | Iloprost | 2.5 | 1.5 | 1 | 40% | 4 |
| Pasquier et al., 2012 (11) | 51 | Male | Upper | Bilateral wrist block with ropivacaine, dexketoprofen | 5 | 0 | 5 | 100% | 2 |
| Poole et al.,  2016 (70) | 47 | Male | Upper | Ibuprofen, topical aloe vera | 1 | 0 | 1 | 100% | 3 |
| Poole et al.,  2016 (70) | 46 | Male | Lower | Iloprost + ibuprofen | 7 | 0 | 7 | 100% | 3 |
| Porter et al.  1976 (95) | 30 | Male | Lower | Reserpine | 36 | 0 | 36 | 100% | 3 |
| Porter et al.  1976 (95) | 30 | Male | Lower | Reserpine | 10 | 0 | 10 | 100% | 3 |
| Poulakidas et al., 2008 (103) | 43 | Male | Lower | Conservative treatment | 0.5 | 0 | 0.5 | 100% | 2 |
| Prommersberger et al., 2001 (86) | 46 | Male | Upper | Acetylsalicylic acid | 3 | 1.5 | 1,5 | 66% | 2 |
| Punja et al.,  1998 (78) | 24 | Male | Lower | Epidural lidocaine | 38 | 38 | 0 | 0% | 4 |
| Orak et al.,  2007 (89) | 18 | Male | Lower | Acetylsalicylic acid, pentoxifylline, LMWH | 13 | 0 | 13 | 100% | 3 |
| Roche-Nagle et al., 2008 (67) | 47 | Male | Upper | Iloprost + ASA | 40 | 40 | 0 | 0% | 4 |
| Russell KW et al., 2013 (104) | 48 | Male | Lower | Conservative treatment | 0.5 | 0 | 0.5 | 100% | 2 |
| Saemi et al.,  2009 (62) | 37 | Male | Upper | tPA + heparin + papaverine | 10 | 0 | 10 | 100% | 3 |
| Santapau et al., 2013 (90) | 35 | Male | Lower | Heparin, ASA | 1 | 1 | 0 | 0% | 2 |
| Shumacker et al., 1964 (12) | 20 | Not specified | Lower | Surgical sympathectomy | 0.5 | 0 | 0.5 | 100% | 2 |
| Shumacker et al., 1964 (12) | 30 | Not specified | Lower | Surgical sympathectomy | 40 | 10 | 30 | 75% | 4 |
| Shumacker et al., 1964 (12) | 44 | Not specified | Lower | Surgical sympathectomy | 20 | 0 | 20 | 100% | 4 |
| Shumacker et al., 1964 (12) | 44 | Not specified | not specified | Surgical sympathectomy | 2.5 | 1 | 1.5 | 60% | 2 |
| Shumacker et al., 1964 (12) | 51 | Not specified | Lower | Surgical sympathectomy | 20 | 1 | 19 | 95% | 4 |
| Shumacker et al., 1964 (12) | 43 | Not specified | Lower | Surgical sympathectomy | 36 | 30 | 6 | 17% | 4 |
| Shumacker et al., 1964 (12) | 60 | Not specified | Upper | Surgical sympathectomy | 10 | 1.5 | 8.5 | 85% | 3 |
| Shumacker et al., 1964 (12) | 25 | Not specified | Lower | Surgical sympathectomy | 3 | 0.25 | 2.8 | 92% | 3 |
| Shumacker et al., 1964 (12) | 38 | Not specified | not specified | Surgical sympathectomy | 10 | 2.5 | 7.5 | 75% | 3 |
| Shumacker et al., 1964 (12) | 63 | Not specified | Lower | Surgical sympathectomy | 36 | 10 | 26 | 72% | 4 |
| Shumacker et al., 1964 (12) | 87 | Not specified | Lower | Surgical sympathectomy | 10 | 0.75 | 9.3 | 93% | 4 |
| Shumacker et al., 1964 (12) | 41 | Not specified | Lower | Surgical sympathectomy | 36 | 10 | 26 | 72% | 3 |
| Shumacker et al., 1964 (12) | 49 | Not specified | Lower | Surgical sympathectomy | 30 | 6.24 | 24 | 79% | 4 |
| Shumacker et al., 1964 (12) | 65 | Not specified | Lower | Surgical sympathectomy | 10 | 10 | 0 | 0% | 3 |
| Shumacker et al., 1964 (12) | 42 | Not specified | Lower | Surgical sympathectomy | 10 | 0.5 | 9,5 | 95% | 3 |
| Shumacker et al., 1964 (12) | 63 | Not specified | Lower | Surgical sympathectomy | 3 | 1 | 2 | 67% | 3 |
| Shumacker et al., 1964 (12) | 75 | Not specified | Lower | Surgical sympathectomy | 1 | 0 | 1 | 100% | 3 |
| Shumacker et al., 1964 (12) | 42 | Not specified | Lower | Surgical sympathectomy | 0.25 | 0 | 0.3 | 100% | 2 |
| Shumacker et al., 1964 (12) | 60 | Not specified | Lower | Surgical sympathectomy | 1 | 1 | 0 | 0% | 3 |
| Shumacker et al., 1964 (12) | 45 | Not specified | Upper | Surgical sympathectomy | 1 | 0 | 1 | 100% | 2 |
| Shumacker et al., 1964 (12) | 45 | Not specified | Upper | Surgical sympathectomy | 0.5 | 0 | 0.5 | 100% | 2 |
| Xiao et al., 2021 (83) | 18 | Female | Upper and lower | Ibuprofen | 20 | 10 | 10 | 50% | 4 |
| Welch et al., 1974 (92) | 21 | Female | Upper and lower | LMWD | 17.5 | 0 | 17.5 | 100% | 3 |

**eTable 2: Patient cohort studies with outcomes quantified by the Hennepin score.**

tPA = Tissue plasminogen activator

| **Article** | **Mean age** | **Sex** | **Extremity involved** | **Grades of injury included** | **Treatment** | **Tissue Salvage Rate ^a^** |
| --- | --- | --- | --- | --- | --- | --- |
| Dole et al., 2018 (33) | 32 | Female: 6  Male: 35 | Lower: 41 | Grade 3 | tPA: | 78% |
|  |  |  |  |  | Conservative: | 43% |
|  |  |  |  |  | Total: | 64% |
| Lindford et al., 2017 (9) | Not specified | 9 cases, Sex not specified | Not specified | Grades 3-4 | tPA: | 77% |
| Nygaard et al., 2017 (24) | 42 | Female: 15  Male: 58 | Upper: 32  Lower: 31  Upper and Lower: 10 | Grades 3-4 | tPA: | 74% |
|  |  |  |  |  | Conservative: | 55% |
|  |  |  |  |  |  |  |

^a^ Taken from the corresponding articles.

**eTable 3: Patient cohort studies with outcomes quantified by digit salvage rate.**

[DR] = digits at risk, [DA] = digits amputated, Digit salvage rate = 1-([DA]/[DR]))*100

ASA = acetylsalicylic acid, LMWD = low molecular weight dextran, HBOT = Hyperbaric oxygen therapy; LMWH = low molecular weight heparin. Parentheses indicate that patients may have been given none, one, or more medications, depending on the preferences of the treating physicians.

| **Article** | **Age** | **Sex** | **Extremity involved** | **Grades of injury included** | **Treatment** | **[DR]** | **[DA]** | **Digit salvage rate** |
| --- | --- | --- | --- | --- | --- | --- | --- | --- |
| Bruen et al., 2007 (32) | Mean: 36 | Female: 7  Male: 25 | Upper: 6  Lower: 20  Upper and Lower: 6 | 3-4 | tPA + heparin: | 59 | 6 | 90% |
|  |  |  |  |  | Conservative treatment: | 234 | 97 | 59% |
|  |  |  |  |  |  |  |  |  |
| Cauchy et al., 2011 (8) | Mean: 33 | Female: 3  Male: 44 | Upper: 18  Lower: 14  Upper and Lower: 15 | 2-4 | ASA + buflomedil + iloprost | 142 | 0 | 100% |
|  |  |  |  |  | ASA + buflomedil + tPA + iloprost | 159 | 5 | 96% |
|  |  |  |  |  | ASA + buflomedil | 106 | 42 | 60% |
| Johnson et al., 2011 (52) | Not specified | 11 cases  Sex not specified | Upper: 9  Lower: 3 | 3-4 | tPA + heparin: | 73 | 43 | 41% |
| Gonzaga et al., 2016 (23) | Mean: 40 | Female: 11  Male: 51 | Upper: 21  Lower: 23  Upper and Lower: 18 | 2-4 | Thrombolysis + papaverine + heparin | 472 | 148 | 69% |
| Tavri et al., 2016 (51) | Mean: 33 | Female: 2  Male: 11 | Not specified | 3-4 | tPA + heparin: | 132 | 26 | 80% |
| Lindford et al., 2017 (9) | Not specified | 9 cases  Sex not specified | Not specified | 3-4 | tPA + papaverine + heparin + ASA + LMWH: | 73 | 24 | 67% |
| Patel et al., 2017 (34) | Mean: 40 | Female: 2  Male: 15 | Not specified | 3-4 | tPA + heparin  (+ nitroglycerin, papaverine, nicardipine, alprostadil): | 80 | 12 | 85% |
|  |  |  |  |  | Conservative treatment: | 100 | 77 | 23% |
| Poole et al., 2021(10) | Mean: 39 | Female: 5  Male: 17 | Upper: 12  Lower: 6  Upper and Lower: 4 | 2-4 | Yukon frostbite protocol | 142 | 29 | 80% |
| Magnan et al., 2021 (18) | Mean: 37 | Female: 4  Male: 54 | Not specified | 3-4 | ASA + Iloprost + HBOT;  ASA + Iloprost: | 177  210 | 3  31 | 98%  85% |
| Wexler et al., 2017 (50) | Mean: 49  Median: 49 | Female: 0  Male: 6 | Upper: 4  Lower: 2 | 3 | tPA (+ heparin, ASA, warfarin): | 45 | 17 | 62% |

**eTable 4: Patient cohort studies with outcomes quantified by phalanx salvage rate.**

[DR] = digits at risk, [DA] = digits amputated, Digit salvage rate = 1-([DA]/[DR]))*100

tPA = Tissue plasminogen activator

| **Article** | **Age** | **Sex** | **Extremity involved** | **Grades of injury included** | **Treatment** | **[PR]** | **[PA]** | **Phalanges salvage rate** |
| --- | --- | --- | --- | --- | --- | --- | --- | --- |
| Paine et al., 2020 (28) | Mean: 34  Median: 31 | Female: 0  Male: 5 | Not specified | 3-4 | tPA + heparin + ASA + LMWH: | 106 | 14 | 87% |
|  |  |  |  |  |  |  |  |  |

**eTable 5: Patient cohort studies with outcomes quantified by amputations.**

tPA = Tissue plasminogen activator, LMWD = Low molecular weight dextran, LMWH = Low molecular weight heparin; Parentheses indicate that patients may have been given none, one or more of the following compounds depending on the preference of the treating physicians.

| **Article** | **Mean age** | **Sex** | **Extremity involved** | **Grades or Degrees of injury included** | **Treatment** | **No Amputation** | | **Amputation** | |
| --- | --- | --- | --- | --- | --- | --- | --- | --- | --- |
|  |  |  |  |  |  | **Nr** | **%** | **Nr** | **%** |
| Carmichael et al., 2022 (57) | Median: 41 | Female: 51  Male 148 | Not specified | Grades 3-4 | tPA + LMWH:  Conservative: | 49  51 | 68 %  40 % | 23  76 | 32 %  60 % |
| Ghumann et al., 2019 (56) | 40 | Female: 4  Male: 18 | Not specified | Degrees 2-4 | HBOT+ NSAID  (+ anti-platelet therapy, anticoagulation): | 14 | 64% | 8 | 36% |
| Jones et al., 2017 (55) | Not specified | 7 cases, sex not specified | Not specified | Grades 3-4 | tPA + heparin (+ anti-platelet therapy, anticoagulation): | 5 | 71% | 2 | 29% |
| Heard et al., 2020 (31) | 44 | Female: 10  Male: 30 | Not specified | Grades 2-4 | tPA + heparin: | 10 | 83% | 2 | 17% |
|  |  |  |  |  | Conservative treatment: | 18 | 64% | 10 | 36% |
|  |  |  |  |  |  |  |  |  |  |
| Woo et al., 2013 (54) | 42 | Female: 4  Male: 13 | Upper: 6  Lower: 6  Upper and Lower: 5 | Degrees 2 – 4 | Talniflumate + alprostadil | 7 | 41% | 10 | 59% |
| Zhang et al., 2020 (53) | 52 | Female: 6  Male: 30 | Lower: 36 | Grades 3 – 4 | Topical rhGM-CSF + alprostadil + Urokinase + Ibuprofen | 19 | 53% | 17 | 47% |
| Zhao et al., 2020 (58) | 44 | Female: 28  Male: 128 | Upper: 60  Lower: 46  Upper and Lower: 50 | Degrees 3  -4 | Alprostadil + Ibuprofen (+ LMWD, Urokinase, LMWH) | 92 | 59% | 64 | 41% |
